# Sol–gel immobilization as a suitable technique for enhancement of α-amylase activity of *Aspergillus oryzae* PP

**DOI:** 10.1080/13102818.2014.947073

**Published:** 2014-10-22

**Authors:** Yana Evstatieva, Mariya Yordanova, Georgi Chernev, Yanislava Ruseva, Dilyana Nikolova

**Affiliations:** ^a^ Faculty of Biology, Department of Biotechnology, Sofia University ‘St. Kliment Ohridski’, Sofia, Bulgaria; ^b^ Faculty of Metallurgy and Material Sciences, Department of Silicate Technology, University Of Chemical Technology and Metallurgy, Sofia, Bulgaria

**Keywords:** *Aspergillus oryzae* PP, amylase, immobilization, sol–gel hybrids

## Abstract

Bioencapsulation of microbial cells in silica-based matrices has proved to be a good strategy to enhance the biosynthetic capabilities and viability of bioproducers. In the present study, mycelium and pellet cultures of strain *Aspergillus oryzae* PP were successfully immobilized in sol–gel hybrid matrices composed of tetraethylorthosilicate as an inorganic precursor, 5% (w/v) starch and 10 or 15% (w/v) polyethylene oxide, or 10% (w/v) calcium alginate as organic compounds. Biosynthetic activity of immobilized cultures was investigated by batch and fed-batch cultivation and the obtained results of 3042.04 IU cm^−3^ were comparable with the enzyme activity of the free cell culture. Immobilized cultures retained their viability and biosynthetic capabilities up to the 744th h during fed-batch fermentation processes. Consequently, sol–gel encapsulation in hybrid matrices could be considered as a promising technique for immobilization of *Aspergillus oryzae* PP in order to increase the α-amylase production.

## Introduction

Amylases, as starch-degrading enzymes, are the most studied group of hydrolases finding application at different sectors of life in both physiological and commercial fields.[1–4] *Aspergillus* species have a great capacity to produce α-amylase which requires the development of a new strategy for increasing the fermentative production of the enzyme.[5]

Cell immobilization is particularly feasible for batch fermentation, because the process is characterized by its easy operation, convenient separation of cells from the broth and high density of the cells.[6] Sol–gel technique is an attractive and effective method for immobilization by building a porous gel network around cells through hydrolysis of tetraalkoxysilanes, giving inert glasses with high porosity and high thermal and mechanical resistance.[7–9]

Several published papers have reported the entrapment of living cells within silica matrices. Yu et al. described a sol–gel method for encapsulation of *Moraxella* spp. cells in porous silicate matrices towards the development of a biosensor.[10] Micro-algae cells of *Chlorella vulgaris* as an example of vegetal cells were also entrapped in sol–gel matrices to preserve the biological activity.[9] Dickson et al. (2009) reported a successful encapsulation of *Synechocystis* sp. PCC 6803 in silica sol–gel matrices and H_2_ production quantities from immobilized cells were comparable to those from cultures in optimized media.[11]

The properties of sol–gel silica materials can be approved by composition of hybrid matrices containing organic compounds such as gelatin, methylmetacrylate, chitosan, polyacrylamide, polyethylene oxide, calcium alginate, etc.[12–15] According to many reports, the association of tough, thermostable, non-swelling inorganic silica materials with soft calcium alginate has several advantages. The gel-forming step is mild enough to maintain the viability and biological activity of the entrapped cells or biomolecules.[16–18] This type of hybrid matrices can also contain organic compounds which are established to be inducing substrates of enzyme activity of interest.

In the present study, mycelium and pellet cultures of strain *Aspergillus oryzae* PP were successfully immobilized in sol–gel hybrid matrices composed of tetraethylorthosilicate as an inorganic precursor, 5% (w/v) starch and 10 or 15% (w/v) polyethylene oxide or 10% (w/v) calcium alginate as organic compounds. The α-amylase activity by the immobilized cells was investigated up to the 864th h during batch and fed-batch fermentation processes and compared with activity of free cell cultures.

## Materials and methods

### Microorganism and fermentation conditions

The fungal strain *Aspergillus oryzae* PP was obtained from the microbial collection of the Department of Biotechnology, Faculty of Biology, Sofia University ‘St. Kliment Ohridski’ (Bulgaria) and used in the present study. The culture was maintained on Sabouro agar at 28 °C.

Mycelium culture was prepared by transferring 20% (v/v) of spore suspension with concentration of 4–8 × 10^6^ spores/cm^3^ into 100 mL of growth medium in 500-mL shake flasks consisting of 0.3% (w/v) NH_4_NO_3_, 0.1% (w/v) KH_2_PO_4_, 0.01% (w/v) KCl, 0.05% (w/v) MgSO_4_.7H_2_O, 0.001% (w/v) FeSO_4_, 1% (w/v) corn steep, 6% (w/v) starch, 4% (w/v) soy flour and 1% (w/v) wheat bran.

Pellet cultures were obtained by 24 h cultivation in liquid medium without wheat bran. The cultivation was carried out at 29 ± 1 °C and 250 r/min, and the mycelia and pellets were used for a fermentation process and sol–gel immobilization in hybrid matrices.

Alpha-amylase production by free and immobilized cultures was carried out at 28–30°C and 250 r/min in 500-mL shake flasks containing 50 mL of Chapek fermentation medium containing of 5% (w/v) starch, 2% (w/v) soy flour and 1% (w/v) wheat bran. 2% (w/v) corn steep was also added to the medium in order to obtain higher enzyme synthesis. Samples were collected for analysis of α-amylase activity up to the 864^th^ h of the fermentation process.

### Immobilization technique

Sol–gel transparent silica hybrid matrices were prepared by substituting part of the inorganic precursor tetraethylorthosilicate with 5% (w/v) starch and 10 or 15% (w/v) polyethylene oxide (PEO) or calcium alginate. The hybrid matrices have been synthesized at room temperature under strictly controlled pH conditions. In all cases the ratio precursor/H_2_O was kept constant and equal to 1. A small amount of 0.1 m HCl was introduced to increase the hydrolysis rate (pH ∼ 1.5). Then a phosphate buffer was rapidly added to raise the pH value to 6.0, suitable for keeping the cell vitality. Different quantities of organic compounds, PEO or calcium alginate, were added to obtain the hybrid material. To obtain one sample, the cell immobilization was carried out using 10 ml mycelium or pellet culture.

### Amylase assay

Alpha-amylase activity was determined spectrophotometrically by the method of Sandstedt, Kneen and Blish (SKB) using 1% (w/v) starch solution as a substrate.[19] One SKB unit of α-amylase activity was defined as the amount of the enzyme which catalyses the hydrolysis of 1 g soluble starch for 1 h at pH −4.7 and at 40°С.

## Results and discussion

Immobilization of microorganisms is considered to be an important tool to improve the cell productivity, but also provides many benefits in comparison with their free forms like increased operational stability, easy separation from the reaction mixture and reusability for industrial applications. Bioencapsulation of microbial cells in silica-based matrices has proved to be a good strategy to enhance the biosynthetic capabilities and viability.

Amylase production by free and immobilized pellet cultures of the fungal strain *Aspergillus oryzae* PP was followed during cultivation processes on rotary shaker at 29 ± 1 °C and 250 r/min. Two organic compounds polyethylene oxide and calcium alginate were used in hybrid matrices composition in order to investigate retention of cell viability and enzyme productivity. The levels of α-amylase obtained were remarkably dependent on the concentration of the organic compound.

**Figure 1.**
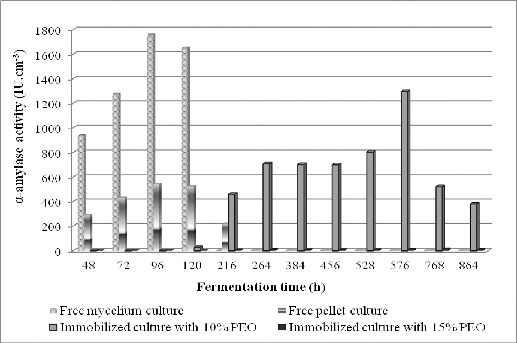
Alpha-amylase activity of strain *Aspergillus oryzae* PP: free mycelium; free pellet culture and pellet culture immobilized in sol–gel matrices with 10% (w/v) and 15% (w/v) polyethylene oxide.

Different concentrations of PEO, as an organic compound of hybrid matrices, were tested for the determination of its effect on the enzyme activity of the investigated strain. Free pellet culture of *Aspergillus oryzae* PP was immobilized in sol–gel hybrid silica materials, composed of tetraethylorthosilicate and two concentrations, 10% and 15% (w/v), of the organic compound PEO. Alpha-amylase activity by free and immobilized cultures was investigated in shake flasks up to the 840th h during the fermentation process (Figure 1).

According to the results, immobilized pellet cultures in sol–gel matrices, containing 10% (w/v) PEO, showed the highest amylase activity of 1297.44 IU cm^−3^ on the 576th h (Figure 1). This activity is higher than the maximal enzyme production of free pellet culture (542.76 IU cm^−3^ at 96th h) and is comparable with α-amylase activity of a free mycelium culture on the 72th h – 1274.76 IU cm^−3^ (Figure 1).

Based on the obtained results, we modified the fermentation medium by the addition of 2% (w/v) corn steep in order to increase the α-amylase production. We received both the enzyme activity of 3011.28 IU cm^−3^ on the 168th h by free cells, cultivated in the optimized medium with the addition of 2% (w/v) corn steep to the fermentation medium, and reduced time of the fermentation process.

Based on this, constructed experiments to study the biosynthetic capabilities of strain *Aspergillus oryzae* PP pellets immobilized in hybrid matrices composed of tetraethylorthosilicate, 5% (w/v) starch and 15% (w/v) polyethylene oxide. Alpha-amylase activity was followed during cultivation processes on a rotary shaker at 29 ± 1 °C and 250 r/min up to 840th h. The biosynthetic activity of free and immobilized pellet cultures showed that the activity of immobilized pellet culture was lower than that of the free mycelium. This growth form of the strain was not suitable to induce higher α-amylase production, because of the ease of separation of pellet biomass from the medium.

**Figure 2.**
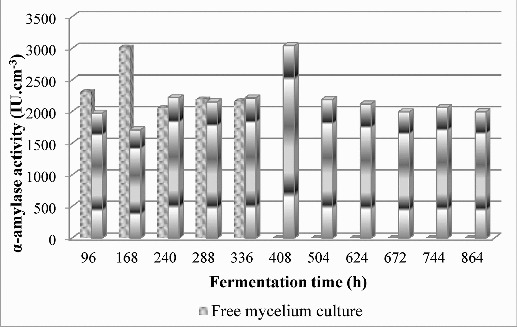
Biosynthesis of α-amylase by free and immobilized mycelium cultures in sol–gel matrices composed of tetraethylorthosilicate, 5% (w/v) starch and 10% (w/v) calcium alginate in optimized fermentation medium.

Based on received data, the remaining experiments were constructed by the use of hybrid matrices containing calcium alginate. One suitable and popular method for cell immobilization is sol–gel encapsulation in hybrid matrices containing calcium alginate. Mycelium cultures of strain *Aspergillus oryzae* PP were immobilized successfully in sol–gel matrices composed of tetraethylorthosilicate, 5% (w/v) starch and 5%, 10% or 15% (w/v) calcium alginate. Alpha-amylase activity by free and immobilized mycelium cultures was investigated in shake flasks up to the 864th h during the fermentation process (Figure 2).

According to the results, α-amylase activity of 3042.04 IU cm^−3^ was obtained from culture immobilized in sol–gel matrix composed of tetraethylorthosilicate, 5% (w/v) starch and 10% (w/v) calcium alginate at the 408th h of the fermentation process. This activity is similar to the α-amylase activity produced by free cell cultures of 3011.28 IU cm^−3^ on the 168th h of the cultivation.

In sol–gel matrix, (5% (w/v) and 15% (w/v) calcium alginate) amylase activity was remarkably lower than the enzyme activity of the free mycelium. We monitored the cell separation and destruction of the hybrid matrix at the end of fermentation process. It is due to substrate diffusion limitations to the interior of the matrix because of lower or higher concentrations of organic compounds.

The use of 10% (w/v) calcium alginate in sol–gel hybrid matrices composition provides an enhancement of productivity of immobilized *Aspergillus oryzae* PP cells. The sol–gel matrix composed of tetraethylorthosilicate, 5% (w/v) starch and 10% (w/v) calcium alginate offers higher porosity for better diffusion of nutrients, which leads to the increase of α-amylase activity and reduces the time of the fermentation processes. This analysis confirmed our previous results about the positive effect of calcium alginate on xylanase enzyme activity (produced by the *Aspergillus awamori* K-1 [20] strain) and the positive effect on the amylase enzyme.[1,3,4] On that basis, we confirmed the positive effect of the immobilization method, applied for the genus *Aspergillus*, as other investigation studies do as well.[21–26]

According to the results immobilized cultures of *Aspergillus oryzae* PP retained their biosynthetic capabilities during long-term fermentation processes. It was found that the method of immobilization had a positive effect on the strain activity.

## Conclusions

In summary, mycelium and pellet cultures of the fungal strain *Aspergillus oryzae* PP were successfully immobilized in hybrid matrices composed of tetraethylorthosilicate as an inorganic precursor, 5% (w/v) starch and 15% (w/v) polyethylene oxide or calcium alginate as organic compounds. The use of 10% (w/v) polyethylene oxide or calcium alginate provided a considerable positive effect on enzyme activity.

The immobilized mycelium cultures with 10% (w/v) calcium alginate show comparable capacity of α-amylase production of free cell culture. The use of calcium alginate as an organic compound in the matrix formation also solved the diffusion problems in the interior of the support. Consequently, sol–gel encapsulation in hybrid matrices could be considered as a promising technique for immobilization of *Aspergillus oryzae* PP in order to increase the α-amylase production.
